# Improving Clinical, Cognitive, and Psychosocial Dysfunctions in Patients with Schizophrenia: A Neurofeedback Randomized Control Trial

**DOI:** 10.1155/2021/4488664

**Published:** 2021-08-12

**Authors:** Renata Markiewicz, Agnieszka Markiewicz-Gospodarek, Beata Dobrowolska, Bartosz Łoza

**Affiliations:** ^1^Psychiatric Nursing Department, Faculty of Health Sciences, Medical University of Lublin, Poland; ^2^Department of Human Anatomy, Faculty of Medicine, Medical University of Lublin, Poland; ^3^Department of Management in Nursing, Faculty of Health Sciences, Medical University of Lublin, Poland; ^4^Department of Psychiatry, Medical University of Warsaw, Poland

## Abstract

**Objectives:**

The aim of this study was to use neurofeedback (NF) training as the add-on therapy in patients with schizophrenia to improve their clinical, cognitive, and psychosocial condition. The study, thanks to the monitoring of various conditions, quantitative electroencephalogram (QEEG) and brain-derived neurotrophic factor (BDNF), was supposed to give an insight into mechanisms underlying NF training results.

**Methods:**

Forty-four male patients with schizophrenia, currently in a stable, incomplete remission, were recruited into two, 3-month rehabilitation programs, with standard rehabilitation as a control group (R) or with add-on NF training (NF). Pre- and posttherapy primary outcomes were compared: clinical (Positive and Negative Syndrome Scale (PANSS)), cognitive (Color Trails Test (CTT), d2 test), psychosocial functioning (General Self-Efficacy Scale (GSES), Beck Cognitive Insight Scale (BCIS), and Acceptance of Illness Scale (AIS)), quantitative electroencephalogram (QEEG), auditory event-related potentials (ERPs), and serum level of BDNF*. Results*. Both groups R and NF improved significantly in clinical ratings (Positive and Negative Syndrome Scale (PANSS)). In-between analyses unveiled some advantages of add-on NF therapy over standard rehabilitation. GSES scores improved significantly, giving the NF group of patients greater ability to cope with stressful or difficult social demands. Also, the serum-level BDNF increased significantly more in the NF group. Post hoc analyses indicated the possibility of creating a separate PANSS subsyndrome, specifically related to cognitive, psychosocial, and BDNF effects of NF therapy.

**Conclusions:**

Neurofeedback can be effectively used as the add-on therapy in schizophrenia rehabilitation programs. The method requires further research regarding its clinical specificity and understanding mechanisms of action.

## 1. Introduction

Schizophrenia is a chronic and relapsing disease characterized not only by the occurrence of delusions and hallucinations but also by the progressive development of cognitive and social deficits [1, 2]. The disease leads to complex and increasing impairments of working memory, concentration, emotions, social achievements, and vocational competences [3–8]. Up to one-half of the patients remain residual or actively psychotic despite optimal pharmacological treatment [9]. Those patients should be offered any of various rehabilitation programs. Optimal treatment of schizophrenia remains a strategic challenge and should integrate various and specific psychosocial interventions in addition to the optimal use of medications [10].

Looking for new, more effective methods of treatment and rehabilitation of schizophrenia, systematic attempts are being made to use neurofeedback (NF) [11]. NF, by allowing patients to perceive and response actively, creates brain stimulation as the cognitive target. That leads to the training of cognitive functions and, ultimately, to the social rehabilitation. Some studies confirm the positive effects of NF therapy, not only in relation to schizophrenia [11, 12] but also to other mental disorders, including anxiety and mood disorders [13, 14], suicidal risk [15–17], ADHD [18], and others [19].

There are various pathophysiological concepts how NF therapies work [11–19]. They refer to neuroplastic and behavioral theories, cognitive training, and conditioning or modulating own neural activity. NF training can be viewed as a form of external influence which uses specific exercises to modify the structure and function of neural networks through learning and memorizing [11]. In 2013, combining functional MRI with NF training, a group of schizophrenia patients was taught to “locate” their brain activity volitionally (in frontal cingulate gyrus) [20, 21]. Repeated stimulation (training) has a positive effect not only on stimulus-response components but also on the change in the intensity of interneuronal connections and the increase in the number of synaptic connections [22].

Regular training contributes to the activity of the brain-derived neurotrophic factor (BDNF), associated with an increase in the expression of neuronal genes and reorganization of synapses [18]. The relationship between activity, including mental activity, and BDNF levels appears to be two-way [23].

Disrupting of BDNF circulation and its downstream signals has been found in many neuropsychological diseases [18]. BDNF is considered to play a major role in the pathogenesis of schizophrenia [24]. The PANSS negative syndrome and PANSS total scores are negatively correlated with the BDNF serum level in patients with schizophrenia [25]. BDNF seems to mediate the antipsychotic effects of ECT and medications [26]. Importantly, the neuropsychological effects of BDNF can be monitored by its peripheral serum level [18, 27, 28]. Together with other neuroproteins, such as the nerve growth factor (NGF) and neurotrophins NT-3 and NT-4/5 (proteins which support the formation of synapses), BDNF is involved in neuronal function [29], growth and differentiation of stem cells, formation of synapses, regulation of neuronal circuits [28], and the formation of memory pathways [30–32]. It is fundamental that neurons must be highly active to respond to growth-promoting action of BDNF [30], which opens the way to therapies such as NF training [24, 30–32].

The aim of this study was to use NF training as the add-on therapy in patients with schizophrenia to improve their clinical, cognitive, and psychosocial condition. The study, thanks to the monitoring of various conditions, QEEG and BDNF, was supposed to give an insight into mechanisms underlying NF training results.

## 2. Methods

### 2.1. Study Design

This study was a randomized, controlled 3-month trial reported with the use of Consolidated Standards of Reporting Trials (CONSORT) guidelines [33]. The trial is registered in the ISRCTN registry (Trial ID: ISRCTN78612833) where the full protocol can be found.

We followed the methods of Markiewicz and Dobrowolska [34] and Markiewicz and Dobrowolska [35]. Forty-four male patients with schizophrenia were assigned to participate in a standard rehabilitation program (group R; N26) or a program of neurofeedback training plus standard rehabilitation (group NF; N18). Both groups continued their treatment as usual in a city day care center, consisted of regular clinical management, psychopharmacotherapy, and standard daily care.

### 2.2. Participants

The inclusion criteria were patients' consent, male gender, clinical diagnosis of schizophrenia [36], age 18–50, right-handedness (writing), no current neurological diseases, mental disability, and neither alcohol nor psychoactive substance addiction. The study was limited to male patients only to reduce the risk of sexual differences in BDNF levels in patients with schizophrenia, affecting cognitive functions and metabolic parameters, as suggested in some other studies [37–39]. BDNF studies involving women require a specific methodology, taking into account the phases of the menstrual cycle [40].

The main group size was set at 60 participants, 30 people for each group. Since we used one or two independent variables in our comparisons, a safe rule of thumb would be a minimum sample size of 2 × 30.

Subjects, after meeting the inclusion criteria, were randomly assigned to two groups, with standard rehabilitation and additional neurofeedback. The allocation to the groups was random (drawing), without the researchers participating in the drawing process and without affecting the final result.

All recruited patients remained stable, i.e., without active psychotic symptoms for not less than 18 months. That is more than a 12-month criterion for “residual schizophrenia” according to ICD-10 [41]. However, patients cannot be treated as a group as “residual schizophrenia.” They were quite young, active, and fit rather the pattern episodic schizophrenia with a progressive or persistent development of “negative” symptoms in the intervals between psychotic episodes [41]. No current suicidal risk was diagnosed. Patients from the R group had on average seven past psychiatric hospitalizations (M 6.67, SD 4.80) and from the NF group eight (M 8.23, SD 7.31). All subjects were administered atypical antipsychotics; they all continued the former treatment (daily dose olanzapine equivalents in milligrams: NF vs. R: M 23.2 SD 7.7 vs. M 22.3 SD 7.0) [42]. None of the patients had taken any anticholinergic drugs.

The mean age of subjects in the R group was 36.38 (SD 8.87) and for the NF group was 37.22 (SD 6.38). The mean body mass index (BMI) in the R group was 29.6 (SD 4.6) and for the NF group was 27.3 (SD 3.2). Most of the patients in both groups smoked cigarettes: R group -69% vs. NF group -61%.

The mean education, calculated in years, for the R group was 13.85 (SD 1.93) and for the NF group was 12.61 (SD 2.85). All other clinical and demographic characteristics were also similar in both groups. Almost all of the patients lived on a disability pension or other social benefits.

### 2.3. Outcome Measures

Clinical (PANSS), cognitive (CTT, d2), psychosocial (BCIS, AIS, and GSES), and electrophysiological parameters (QEEG_NF) as well as BDNF levels were examined with the following:

1. Positive and Negative Syndrome Scale (PANSS) [43]: for measuring symptoms, syndromes, and general severity of schizophrenia
2. Color Trails Test (CTT) [44]: part 1 (CTT-1) to assess visual performance and psychomotor speed, when connecting numbers in a string from 1 to 25 and part 2 (CTT-2) to assess performance skills and working memory, when connecting numbers with simultaneous selection of a color sequence in a string from 1 to 25
3. d2 test of attention (d2) [45]: to measure processing speed (amount of material processed in a specific time), quality of work (accuracy and the errors made), and persistence as an indicator of features of behavior manifested during work (irritation, stability of work or lack of it, discouragement, and fatigue); the level of concentration was a result of the interaction of these behaviors and a product of the stimulus and control coordination
4. Beck Cognitive Insight Scale (BCIS) [46]: to evaluate patients' self-reflectiveness and their self-certainty in their interpretations of their experiences
5. Acceptance of Illness Scale (AIS) [47]: to estimate limitations imposed by the illness, lack of independence due to the illness, and reduction of self-esteem
6. General Self-Efficacy Scale (GSES) [47]: to check patients' ability to cope with stressful or challenging social demands, relying on their self-efficacy
7. Brain-derived neurotrophic factor (BDNF): serum level of BDNF was determined following blood sampling into a clot tube using a noncontact method; laboratory levels were determined immunoenzymatically with ELISA technique
8. Quantitative electroencephalography-neurofeedback (QEEG-NF) [48]: to map and statistically meta-analyze EEG recordings in relation to neurofeedback stimulation

### 2.4. Procedures and Equipment

The NF training sessions were held twice a week for three months. The training scheme assumed the gradation of task difficulty, taking into account the individual progress of the workshops. The galvanic skin response (GSR) method was used. GSR has two components, the general tonic-level electrodermal component (skin conductance level, SCL) and the phasic component (skin conductance responses, SCRs), which are indispensable diagnostic parameters in the management of mental disorders. They can be used as reference in GSR-NF to modulate the patient's emotional state depending on the current needs [49]. The GSR-NF training sessions were conducted in the CENTER (relaxation), BALANCE (concentration), and INSECTS (self-control) modules using a DigiTrack apparatus (EEG-DigiTrack Biofeedback-EEG+SpO_2_+HR).

Quantitative EEG (QEEG) analysis was performed using an Elmiko Medical Company, Poland, Warsaw. Patients were fitted with two electrodes in the central region (F-z, C-z). Fast Fourier transform (FFT) algorithm transformed the raw EEG frequency recording into the so-called QEEG power spectrum. Rhythm evaluations from the selected areas were performed twice in both groups, at the beginning of the experiment and after 3 months [48].

NF trainings were performed in accordance with the approved schedule; training was conducted in a soundproof room always at a specified time, mainly after the breakfast. Patients were requested not to drink coffee or smoke for one hour before the training. The measurements were made by the exosomatic method with DC (direct current) using electrodes placed on the index and ring fingers of the left hand and connected to the device displaying the successive training modules. The training exercises in the individual modules were presented on the monitor screen, and the patient performed them in accordance with the instruction. The task of the subjects performing the CENTER exercise, in which they had to bring bubbles appearing on the screen into a circle in the center of the screen, was to achieve relaxation, especially by controlling/modulating their breath and heart rate. The greater the relaxation, the faster the patient performed the task and went to the next level of the module. Training in the BALANCE module was designed to improve concentration. The subjects' task was to achieve a state of maximum concentration, as they placed and balanced a ball in the middle of a tilting board. The task in the INSECT module was to reach a state of internal balance between cognitive and executive functions. The subjects had to recognize moving and hidden insects on the monitor screen and click on them with the mouse. The slow movement of the insects reflected gradual achievement of internal balance during training, which made it easier for the patient to complete the task. The GSR apparatus registered the neurophysiological changes which determined the subjects' psychophysical condition, on the basis of their skin resistance, which was the result of internal tension and stress. In situations of increased tension and stress, the autonomic system caused a number of changes in the body, including increased secretion of sweat, which is a type of conductor. In this situation, immunity decreased. The opposite was true when internal tension and stress were absent or at low levels. The conductor, which was sweat, occurred at a low level, resulting in an increase in resistance (stabilization of the psychophysical state). The recorded resistivity data allowed assessment of the neurophysiological state of the subject, which was presented in the form of a resistivity curve and QEEG data. In this situation, the resistance decreased. The opposite was true when internal tension and stress were absent or at low levels. The conductor that was sweat occurred at a low level, resulting in an increase in resistivity (stabilization of the psychophysical state). The recorded resistivity data allowed assessment of the neurophysiological state of the subject, which was presented in the form of a resistivity curve and QEEG data. Before each patient examination, the DigiTrack instrument was tested for technical performance [50].

The training time was set by the computer program and was 5 min for the CENTER and BALANCE modules and 10 min for the INSECT module. At the end of each session, the patient's results were recorded graphically. Prior to NF training and after a 3-month program, the level of clinical, cognitive, and social deficits was assessed. Long-term NF programs are warranted in research [51, 52].

The standard rehabilitation consisted in enriching the daily routine with social activities that building up team competences, playing social roles, personal acceptance, and growing own independence. At least one teamwork session was offered daily.

The EEG potentials were tested using a Cognitrace apparatus. Twenty-one cup electrodes (an international 10–20 electroencephalogram system with ear electrodes, ground, and reference) (Fp-z, F-z, C-z, P-z, O-z, Fp1, Fp2, F3, F4, C3, C4, P3, P4, O1, O2, F7, F8, T3, T4, T5, T6), two ear electrodes A1 and A2, and GND were attached to the patient's head. The patient stayed in a separate, dark room. The test was performed with the subject in a sitting position, with eyes closed, and wearing earphones through which the acoustic stimuli were delivered in accordance with the oddball paradigm (a series of tones with frequencies in the range from 1000 Hz to 2000 Hz of ca. 70 dB were presented for ca. 100 ms in a random sequence). The P300 test, determining exogenous cognitive potential, was performed twice. One test lasted 3 min and 20 sec and contained 80% of frequent stimuli and 20% of rare (important) stimuli. The subject was required to respond to the rare stimuli by pressing the button. The measurements were performed twice. QEEG was performed in each patient, three months apart, before and after program. The patients had two electrodes placed on their heads, in the F-z and C-z regions, and the fast Fourier transform (FFT) algorithm transformed the raw EEG recording into frequencies for statistical processing (the so-called QEEG power spectrum). In the studied group, the brain rhythm from the two selected areas was evaluated twice [51].

### 2.5. Statistical Analyses

The values of the investigated variables were presented as the means and standard deviations. The sociological and demographic parameters were presented as numbers and percentages. The results were compared using Student's *t*-test for dependent samples, nonparametric Mann-Whitney *U*-test, and Spearman's rank correlation coefficient. Differences were considered to be statistically significant at *P* < 0.05. Analyses were performed using Statistica 13.3.

### 2.6. Ethical Issues

The study protocol was approved by the local Bioethics Committee with approval no. KE-0254/35/2016. All the patients invited to take part in the study gave their written informed consent.

## 3. Results

The baseline and 3-month final results of combined neurofeedback/rehabilitation (NF) versus standard rehabilitation (R) programs are presented in Table 1. For QEEG only, results significantly different were presented. The other QEEG coefficients around C-z and F-z (SMR/beta2—tension and stress factor, alpha/SMR—sensory and motor activity factor, alpha/beta—executive function index, and beta/alpha—thinking and action factor) were not statistically significant.

As both groups, combined NF/rehabilitation and standard rehabilitation, led to statistically significant changes, the next analyses were made to determine which form of therapy was more effective. For this purpose, analyses were performed between the groups in the magnitude of change of pre- and posttherapy results. Only two of the primary outcomes, i.e., BDNF and GSES, were significantly different between groups, depicting NF training advantages over standard rehabilitation program (Table 2).

Looking for significant relationships between clinical data (PANSS) and biochemical (BDNF) as well as electrophysiological effects (QEEG, ERPs) in the group R, the correlation matrix is performed in Table 3.

Correlation analysis showed that in the group R, standard rehabilitation reduced the severity of positive and negative symptoms of PANSS, what was associated with QEEG theta/SMR ratio (known for improvement in the patients' attention and concentration), as well as confirmed by the shortened P2 latency (known for selective attention and mental representations). Similar correlation matrix was made for clinical data (PANSS), biochemical (BDNF), and electrophysiological effects (QEEG, ERPs) in the NF group (Table 4).

Correlation analysis conducted in the NF group showed that NF training reduced the severity of negative and general syndromes measured by PANSS, improved attention and concentration (QEEG), and shortened P2 latency what seems to be beneficial for selective attention.

### 3.1. Post Hoc Analyses

#### 3.1.1. PANSS

Since the in-between group differences for PANSS syndromes were close to significance on behalf of the NF group, we verified post hoc loadings of what symptoms may constitute those clinical effects. The clinical improvement is driven mostly by reductions of the following:

1. P2: conceptual disorganization (*P* ≤ 0.001, *t* = −5.66)
2. N2: emotional withdrawal (*P* ≤ 0.05, *t* = −2.05)
3. N6: lack of spontaneity and flow of conversation (*P* ≤ 0.02, *t* = −2.65)
4. G8: uncooperativeness (*P* ≤ 0.01, *t* = −2.72)
5. G11: poor attention (*P* ≤ 0.05, *t* = −1.96)
6. G16: active social avoidance (*P* ≤ 0.01, *t* = −2.83)

The above set of symptoms includes PANSS subscales related to cognitive and psychosocial functioning. The validity and homogeneity of this domain should be verified in a sufficiently large factor study.

#### 3.1.2. GESE

Post hoc analyses of GESE subscales were performed to approximate the essence of statistically significant changes of total results on behalf of the NF group. Differences before and after therapy were significant in two subscales, i.e.,

1. #1 “Manage to solve difficult problems if I try hard enough” (*P* ≤ 0.04, *t* = 2.09)
2. #3 “Stick to my aims and accomplish my goals” (*P* ≤ 0.03, *t* = 2.33)

Both subscales reflected the increase in decision-making efficacy of the NF group.

## 4. Discussion

Our study arose from a basic need of more effective treatment of schizophrenia. NF training is a relatively new, noninvasive therapeutic method that is being tested in current schizophrenia studies and gives hope for improving the effectiveness of its therapy [24].

This clinical trial proved that rehabilitation programs, especially add-on NF/rehabilitation programs, are not only the vehiculum for cognitive and psychosocial stimulation but also have the potential to improve more general symptoms and syndromes of schizophrenia (Table 1). NF training, based on classical feedback between the behavioral activity and neurophysiological functioning, made it possible to obtain results going beyond simple compensation of disease deficits. Add-on NF training could not only enhance the rehabilitation scores but also the results of overall treatment.

The primary outcomes of this study were in-between group differences in pre- and posttherapy results (Table 2). Two variables turned out to be significantly different, i.e., BDNF and GSES scores. The magnitude of these changes may prove the benefits of add-on NF training compared to standard rehabilitation alone.

The posttherapy increasement of BDNF was observed in both the R and NF groups; however, the NF group score was significantly higher than the result of the R group (Table 2). Moreover, in the add-on NF group, higher BDNF serum level was significantly and negatively correlated with all clinical syndromes of PANSS (positive, negative, general, and its total score) (Table 4), while such correlations were not confirmed in the R group (Table 3).

Several studies unveiled an inverse correlation between the BDNF level and the PANSS-negative subscale [53–55]. In our work, we confirmed a broader relationship of all subscales and the total PANSS score with the BDNF level. Our patients were not residual, rather consolidating remission with active treatment, contrary to other studies including mostly older, residual patients, with little variability of clinical status. However, in a study of Zhang et al. involving younger patients treated for exacerbation of schizophrenia, BDNF levels were closely and inversely associated with improvements in all subscales and the total PANSS score, as we observed [56]. More unique finding of our study is that add-on neurofeedback—and not just any active treatment or standard rehabilitation—has a specific effect on increasing BDNF level and clinical improvement in schizophrenia. Post hoc analyses unveiled a hypothetical factor, reflecting specifically add-on NF training, that could be extracted from PANSS results. However, to make it validated, number of participants should be multiplied for factor analysis.

Other determinants, potentially affecting BDNF level in patients with schizophrenia, like age, gender, BMI, smoking, antipsychotic generation (all atypical), and doses, were not different or statistically different between the R and NF groups [57]. All these factors can significantly modify the level and activity of BDNF in association with the cytokine system [57, 58]. Residual schizophrenia, and its most known manifestation (cognitive impairment), is characterized by a typical biochemical decrease in BDNF and TNF-*α*, and at the same time an increase in IL-2, IL-6, and IL-8 [58]. Since resources for direct impact on this system are limited, the BDNF increase induced by specific add-on NF rehabilitation program opens up potential regulatory opportunities.

During the study, a number of cognitive parameters improved in both the NF and R groups, but in the primary comparison of pre- and posttherapy results, the GSES results mostly demonstrated the benefits of add-on NF training compared to standard rehabilitation. In-depth post hoc analyses of the GSES results indicated that patients from the NF group achieved higher self-efficacy, easier problem-solving, and more effective planning and sticking to the goals, compared to the R group. GSES is a validated tool for screening psychopathological disorders, where “positive results” correlate with increased motivation, optimism about achieving goals, and “negative results” correlate with low mood, anxiety, and stress. That means the improvement of GSES results in our study reflects practically a one-dimensional shift from patients' inactivity to their activity, strengthening coping capacity, and adaptation ability [59]. The relationship between both primary outcomes, BDNF and GSES, can be explained according to Nieto et al. positioning BDNF as a universal biomarker of cognition in schizophrenia, reflecting its different stages and origins [60]. However, for Hori et al., coefficients of correlation between BDNF levels and cognitive dysfunctions are too small and may not be considered as neurocognitive biomarkers for schizophrenia [54].

Similar to our work, Iwata et al. comparing the effects of standard rehabilitation and computer training based on NF proved that the group training with the COGPACK application (Japanese version) achieved a significantly greater improvement in cognitive processing, as confirmed by the Brief Assessment of Cognition in Schizophrenia (BACS) and the Life Assessment Scale for the Mentally Ill (LISMI) [61]. Also, Surmeli et al. using QEEG training in rehabilitation patients with schizophrenia established that neurofeedback can improve cognitive results [62]. The significant reduction (about 20%) of positive and negative symptoms (PANSS) was obtained and accompanied by changes of QEEG pattern, ultimately different from that found in chronic schizophrenia. Perhaps the most important was that the long-term persistence of beneficial effects in cognitive domains was demonstrated stable in a 22-month follow-up with a majority (4/5) of participants [62].

Beyond group differences in pre- and posttherapy results, we found that add-on neurofeedback training (NF group) generated significant effects in nearly all dependent domains. The differences were found in clinical scores (PANSS) and psychosocial (BCIS A and A-B, AIS, and GSES), biochemical (BDNF), and electrophysiological results (QEGGs, ERPs). Thanks to that, patients gained substantial improvement in such domains like reflectiveness (BCISS), illness acceptance (AIS), and higher performance of self-efficacy (GSES). Electrophysiological effects, as demonstrated by theta/beta and theta/SMR scores, gave the prospect of improving the efficiency of working memory and especially patients' concentration. The N1-P2 complex score, much improved in the NF group, may be interpreted as highly beneficial in relation to the improvement of signal gating and the schizophrenia concept as an information metabolism disorder [59].

Contrary to that, statistically significant differences in the R group were found only (apart from PANSS results) in d2 scores - in the reduction in the number of errors, the subjects made during d2 test and the improvement in their concentration ability. However, d2 scores were not significantly different between R and NF groups.

## 5. Conclusions

1. The neurofeedback training can strengthen and extend the scope of clinical, cognitive, and psychosocial rehabilitation of schizophrenia patients
2. Specifically and significantly, patients' BDNF serum level and identifying themselves as competent to act (self-sufficiency) can be increased combining neurofeedback training with rehabilitation versus standard rehabilitation alone
3. The neurofeedback training induces pattern of significant changes of electrophysiological potentials (QEEG, ERPs), which is correlated with improvement of positive, negative, and general symptomatology of schizophrenia
4. The overall results should be interpreted with caution due to limitations in the size of the study group and gender of the participants (only males). Moreover, longitudinal study is recommended in order to analyze maintenance of the effect of neurofeedback training

## Figures and Tables

**Table 1:** Baseline and final results of combined NF/standard rehabilitation versus standard rehabilitation.

Variable	Group	Baseline		Final		Difference absolute	Difference significance		Confidence level	
		M	SD	M	SD		*T*	*P*	−95%	+95%
PANSS-POS	NF	9.06	2.04	7.50	2.23	1.56	10.719	**0.001**	−1.86	−1.25
	R	9.28	2.01	8.24	2.01	1.04	6.186	**0.001**	0.69	1.39

PANSS-NEG	NF	13.94	3.92	11.83	4.48	2.11	8.304	**0.001**	−2.65	−1.57
	R	15.16	3.51	14.08	4.47	1.08	2.596	**0.016**	0.22	1.94

PANSS-GEN	NF	24.83	3.35	22.61	3.71	2.22	10.736	**0.001**	−2.66	−1.79
	R	27.44	3.31	25.88	4.20	1.56	2.742	**0.011**	0.39	2.73

PANSS-TOT	NF	47.83	8.49	41.94	9.64	5.89	11.834	**0.001**	−6.94	−4.84
	R	51.92	7.22	48.20	9.36	3.72	3.375	**0.003**	1.45	6.00

CTT-1	NF	57.19	26.16	49.31	24.92	7.88	1.865	0.082	-16.88	1.13
	R	59.50	24.00	54.29	19.74	5.21	1.861	0.076	-0.58	11.00

CTT-2	NF	121.50	55.62	109.06	42.44	12.44	1.535	0.146	-29.71	4.83
	R	120.58	37.64	110.33	32.59	10.25	1.725	0.098	-2.04	22.54

d2. %B (errors)	NF	10.70	11.09	8.71	10.33	1.99	0.741	0.471	−7.74	3.77
	R	9.01	10.89	6.25	7.01	2.76	2.107	**0.046**	0.05	5.47

d2. ZK (ability to concentrate)	NF	99.06	44.59	105.94	47.66	6.88	−0.985	0.340	−8.00	21.75
	R	107.42	46.69	117.88	35.71	10.46	−2.078	**0.049**	−20.87	−0.05

BCIS A (self-reflectiveness)	NF	22.72	4.80	25.72	3.14	3.00	−3.170	**0.006**	−5.00	−1.00
	R	21.15	4.47	22.12	4.96	0.96	−0.911	0.371	−3.14	1.21

BCISS A-B (composite index)	NF	8.78	5.35	12.22	3.17	3.44	−2.946	**0.009**	−5.91	−0.98
	R	6.38	4.83	7.35	5.12	0.96	−1.000	0.327	−2.94	1.02

AIS (illness acceptance)	NF	22.67	8.95	26.44	6.46	3.78	−2.547	**0.021**	0.65	6.91
	R	25.96	8.77	25.00	7.83	0.96	0.557	0.583	−2.60	4.52

GSES (self-efficacy)	NF	23.78	5.43	27.61	5.09	3.83	−3.239	**0.005**	1.34	6.33
	R	30.15	5.76	28.69	6.16	1.46	1.000	0.327	−1.55	4.47

BDNF serum	NF	44.78	10.69	55.50	10.76	10.72	−6.185	**0.001**	7.06	14.38
	R	50.16	11.38	52.96	10.70	2.80	−1.575	0.128	−6.47	0.87

QEEG C-z theta/beta	NF	1.92	0.57	2.29	0.88	0.37	−2.632	**0.018**	0.07	0.67
	R	2.35	0.94	2.49	0.82	0.14	−1.453	0.159	−0.34	0.06

QEEG F-z theta/SMR	NF	2.07	0.64	2.37	0.80	0.30	−2.358	**0.031**	0.03	0.57
	R	2.49	1.00	2.60	0.83	0.10	−1.013	0.321	−0.31	0.10

F-z N1 (amplitude)	NF	−3.95	2.53	−5.36	1.93	1.41	2.588	**0.020**	−2.57	−0.26
	R	−5.29	3.93	−6.58	3.44	1.30	1.263	0.219	−0.83	3.42

C-z P2 (latency)	NF	208.82	14.81	196.06	18.27	12.77	2.643	**0.018**	−23.01	−2.52
	R	203.92	23.94	205.04	21.70	1.13	−0.185	0.855	−13.68	11.43

M: mean value; SD: standard deviation; CV%: coefficient of variation; *T*: Student's *t*-test; *P*: level of significance; PANSS-POS: Positive and Negative Syndrome Scale-Positive; PANSS-NEG: Positive and Negative Syndrome Scale-Negative; PANSS-GEN: Positive and Negative Syndrome Scale-General; PANSS-TOT: Positive and Negative Syndrome Scale-Total; BDNF: brain-derived neurotrophic factor; BCIS: Beck Cognitive Insight Scale; AIS: Acceptance of Illness Scale; GSES: General Self-Efficacy Scale; QEEG C-z theta/beta: attention factor of the central area; QEEG F-z theta/SMR: concentration factor of the central area; F-z N1 (amplitude): amplitude of the first negative component of the central area; C-z P2 (latency): delay of the second positive component of the central area.

**Table 2:** Primary outcomes: statistically significant differences between groups R and NF in the magnitude of change from pre- to posttherapy outcomes.

Variable (change between measurements for the variable)	Group R		Group NF		In-between comparisons	
	M	SD	M	SD	*t*^A^/*U*^B^	*P*
BDNF (neurotrophic factor)	2.80	8.89	10.72	7.35	−3.093^A^	0.004
GSES (self-efficacy)	−1.46	7.45	3.83	5.02	119.5^B^	0.005

M: mean; SD: standard deviation; A: Student's *t*-test; B: Mann-Whitney *U* test; *P*: statistical significance.

**Table 3:** Correlations between the magnitude of changes from pretherapy to posttherapy measurements in the group of patients following a standard rehabilitation program (group R).

Variable	Group R (standard rehabilitation program)								
	PANSS-POS	PANSS-NEG	PANSS-GEN	PANSS-TOT	BDNF (ng/ml)	QEEG theta/beta	QEEG theta/SMR	N1 amplitude	P2 latency
PANSS-POS	*—*	*0.315*	*0.588* ^∗^	*0.712* ^∗^	*−0.211*	*0.034*	*0.078*	*0.193*	−*0.405*
PANSS-NEG	*0.315*	*—*	*0.568* ^∗^	*0.799* ^∗^	−*0.343*	*0.388*	*0.399*	−*0.028*	−*0.100*
PANSS-GEN	*0.588* ^∗^	*0.568* ^∗^	*—*	*0.880* ^∗^	−*0.085*	*0.038*	*0.042*	*0.272*	−*0.030*
PANSS-TOT	*0.712* ^∗^	*0.799* ^∗^	*0.880* ^∗^	*—*	−*0.221*	*0.147*	*0.168*	*0.192*	−*0.174*
BDNF (ng/ml)	−*0.211*	−*0.343*	−*0.085*	−*0.221*	—	0.101	0.140	0.433	−0.117
QEEG theta/beta	*0.034*	*0.388*	*0.038*	*0.147*	0.101	—	0.904^∗^	0.007	−0.498^∗^
QEEG theta/SMR	*0.078*	*0.399*	*0.042*	*0.168*	0.14	0.904^∗^	—	0.181	−0.488^∗^
N1 (amplitude)	*0.193*	−*0.028*	*0.272*	*0.192*	0.433	0.007	0.181	—	−0.555^∗^
P2 (latency)	−*0.405*	−*0.100*	−*0.030*	−*0.174*	−0.117	−0.498^∗^	−0.488^∗^	−0.555^∗^	—

Correlations were assessed using Spearman's rank correlation coefficients (italics) and Pearson's *r*; statistically significant correlations (*P* < 0.050) are marked with an asterisk (∗).

**Table 4:** Correlations between the magnitudes of changes from pretherapy to posttherapy measurements in the group of patients participating in a combined NF/standard rehabilitation program (group NF).

Variable	Group NF (combined NF/standard rehabilitation)								
	PANSS-POS	PANSS-NEG	PANSS-GEN	PANSS-TOT	BDNF (ng/ml)	QEEG theta/beta	QEEG theta/SMR	N1 amplitude	P2 latency
PANSS-POS	*—*	*0.737* ^∗^	*0.851* ^∗^	*0.877* ^∗^	−*0.770*^∗^	*0.171*	*0.274*	−*0.106*	*0.074*
PANSS-NEG	*0.737* ^∗^	*—*	*0.846* ^∗^	*0.920* ^∗^	−*0.857*^∗^	*0.004*	*0.018*	−*0.228*	−*0.061*
PANSS-GEN	*0.851* ^∗^	*0.846* ^∗^	*—*	*0.956* ^∗^	−*0.804*^∗^	*0.112*	*0.169*	−*0.103*	*0.172*
PANSS-TOT	*0.877* ^∗^	*0.920* ^∗^	*0.956* ^∗^	*—*	−*0.832*^∗^	*0.149*	*0.209*	−*0.166*	*0.061*
BDNF (ng/ml)	−*0.770*^∗^	−*0.857*^∗^	−*0.804*^∗^	−*0.832*^∗^	—	0.123	0.057	0.153	0.296
QEEG theta/beta	*0.171*	*0.004*	*0.112*	*0.149*	0.123	—	0.875^∗^	−0.161	−0.127
QEEG theta/SMR	*0.274*	*0.018*	*0.169*	*0.209*	0.057	0.875^∗^	—	−0.298	−0.297
N1 (amplitude)	−*0.106*	−*0.228*	−*0.103*	−*0.166*	0.153	−0.161	−0.298	—	0.035
P2 (latency)	*0.074*	−*0.061*	*0.172*	*0.061*	0.296	−0.127	−0.297	0.035	—

Correlations were assessed using Spearman's rank correlation coefficients (italics) and Pearson's *r*; statistically significant correlations (*P* < 0.050) are marked with an asterisk (∗).

## Data Availability

All data is available with the authors on reasonable request.

## Abbreviations

GSR: Galvanic skin response
fMRI: Functional magnetic resonance
QEEG: Quantitative changes in the EEG (theta/beta ratio, theta/SMR ratio)
BDNF: Brain-derived neurotrophic factor
AIS: Acceptance of Illness Scale
GSES: Self-Efficacy Scale
BCIS: Beck Cognitive Insight Scale
CTT-1: Test of frontal dysfunction
CTT-1: Test of visual performance and psychomotor speed
CTT-2: Test of frontal dysfunction
CTT-2: Test of performance skills and working memory
d2 psychological test: Test of attention
ERP: Event-related potentials
N1: First negative-going component
P2: Second positive-going component
QEEG: Quantitative EEG
PANSS: Positive and Negative Syndrome Scale
F-z: Frontal brain region
C-z: Central brain region
CNS: Central nervous system
SCRs: Skin conductance responses.

## Supplementary Materials

Supplementary Materials CONSORT 2010 checklist of information to include when reporting a randomized trial.
