# Supplementary material for: Improving Clinical, Cognitive, and Psychosocial Dysfunctions in Patients with Schizophrenia: A Neurofeedback Randomized Control Trial
Source: Neural Plast. 2021 Aug 12;2021:4488664. doi: 10.1155/2021/4488664 (PMC8380506; doi:10.1155/2021/4488664)
Supplement: Supplementary Materials — CONSORT 2010 checklist of information to include when reporting a randomized trial. [file 4488664.f1.zip › CONSORT Flow Diagram.docx]

**
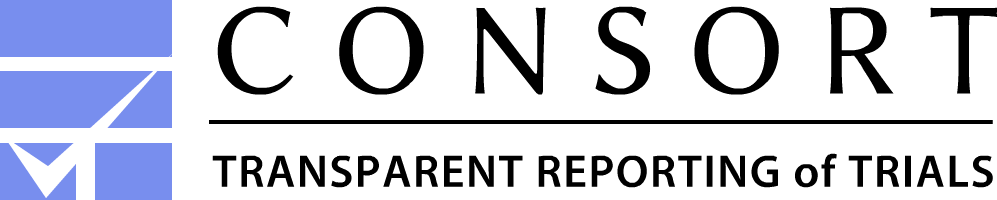
**

**CONSORT 2010 Flow Diagram**

Allocated to intervention (n=30)

♦ Received allocated intervention (n=30)

♦ Did not receive allocated intervention (n=0)

Allocated to intervention (n=30)

♦ Received allocated intervention (n=30)

♦ Did not receive allocated intervention (n=0)

- Lost to follow-up (n=5)
- Discontinued intervention (n=7, withdrawal of consent)
- Lost to follow-up (n=1)
- Discontinued intervention (n=3, withdrawal of consent)
- Analysed (n=18)
- Excluded from analysis (n=0)
- Analysed (n=26)
- Excluded from analysis (n=0)

## Follow-Up

## Analysis

## Enrollment

## Allocation

Randomized (n=60)

Excluded (n=10)

♦  Not meeting inclusion criteria (n=6)

♦  Declined to participate (n=3)

♦  Other reasons (n=1)

Assessed for eligibility (n=70)
